# Classification of mathematical test questions using machine learning on datasets of learning management system questions

**DOI:** 10.1371/journal.pone.0286989

**Published:** 2023-10-18

**Authors:** Gun Il Kim, Sungtae Kim, Beakcheol Jang

**Affiliations:** 1 Graduate School of Information, Yonsei University, Seoul, South Korea; 2 ABLE EduTech Inc., Seoul, South Korea; Virtual University of Pakistan, PAKISTAN

## Abstract

Every student has a varied level of mathematical proficiency. Therefore, it is important to provide them with questions accordingly. Owing to advances in technology and artificial intelligence, the Learning Management System (LMS) has become a popular application to conduct online learning for students. The LMS can store multiple pieces of information on students through an online database, enabling it to recommend appropriate questions for each student based on an analysis of their previous responses to questions. Particularly, the LMS manages learners and provides an online platform that can evaluate their skills. Questions need to be classified according to their difficulty level so that the LMS can recommend them to learners appropriately and thereby increase their learning efficiency. In this study, we classified large-scale mathematical test items provided by ABLE Tech, which supports LMS-based online mathematical education platforms, using various machine learning techniques according to the difficulty levels of the questions. First, through t-test analysis, we identified the significant correlation variables according to the difficulty level. The t-test results showed that answer rate, type of question, and solution time were positively correlated with the difficulty of the question. Second, items were classified according to their difficulty level using various machine learning models, such as logistic regression (LR), random forest (RF), and extreme gradient boosting (xgboost). Accuracy, precision, recall, F1 score, the area under the curve of the receiver operating curve (AUC-ROC), Cohen’s Kappa and Matthew’s correlation coefficient (MCC) scores were used as the evaluation metrics. The correct answer rate, question type, and time for solving a question correlated significantly with the difficulty level. The machine learning-based xgboost model outperformed the statistical machine learning models, with a 85.7% accuracy, and 85.8% F1 score. These results can be used as an auxiliary tool in recommending suitable mathematical questions to various learners based on their difficulty level.

## Introduction

Mathematics is the most fundamental subject taught in elementary schools because it helps in developing critical thinking and logical reasoning. It is important to know that every student has a different set of skills and proficiency for learning mathematical concepts and that they are provided with questions in accordance with their individual abilities. Owing to advancements in technology and artificial intelligence, the most popular way of interacting and communicating with students through online learning is using the Learning Management System (LMS). This system uses an online database to store information on students. Thus, it can recommend appropriate questions to each student based on previous questions they have solved. The LMS is an online learning platform that can systematically evaluate and monitor learners’ skills [1].

It is important to ask learners appropriate questions to evaluate their abilities. In numerous previous studies, questions were classified after considering various variables, ensuring that the most suitable question would be suggested next for the learner. Jayalakshmi and Sheshasaayee [2] and Ray et al. [3] explained that questions could be classified according to syntax, vocabulary, and context using machine learning techniques. Fei et al. [4] used a deep learning-based artificial neural network (ANN) model to classify question difficulty levels by matching the similarity of keywords of items based on the frequency of words and the length of each question. Similarly, Ahmed and Anto [5] used a support vector machine (SVM) model [6] to analyze the question keywords before developing and introducing an automatic question-answering system. Kim [7] developed an algorithm for classifying multiple-choice questions based on a learning dataset developed in a computer programming course for students.

However, most previous studies on question classification have focused on small-scale datasets using the conventional method of analysis to accompany their experimental and theoretical assumptions. Although their theories are significant and applicable for small sets of data, they become ineffective when applied to large-scale data because they require the incorporation of a more advanced algorithm to classify more complex questions. Unlike the limitations of using statistical analysis on large-scale data, machine-learning models are more powerful and capable of solving high-dimensional problems that are difficult for humans to resolve. Therefore, there is an urgent need to classify large-scale questions using an improved method for classifying problems based on machine learning.

In this study, we used various machine learning models, such as logistic regression (LR) [8], random forest (RF) [9], and extreme gradient boosting (xgboost) [10], to classify large-scale mathematical test items provided by ABLE Tech, one of the companies that provides LMS-based online mathematical education platforms. To determine the main features that affected the difficulty level of questions, we initially set up a t-test validation for feature selection. Using the most-correlated variables in machine learning models is essential to their performance; the application of irrelevant features can result in drastic drops in performance. To improve performance reliability, we evaluated our models using seven evaluation metrics: accuracy, precision, recall, F1 score, AUC-ROC, Cohen’s Kappa, and Matthew’s Correlation Coefficient score.

### Contribution

The main contributions of this study are given as follows:

1. The most relevant features found through the statistical t-test analysis were answer rate, question type, and solving time; these variables correlated most significantly with the difficulty level.
2. To compare the performance of the several machine learning models more reliably, we considered seven evaluation metrics.
3. The xgboost model outperformed the other machine-learning models in the multi-class classification problem with the maximum accuracy, F1-score, Kappa and MCC scores.

### Paper organization

The remainder of this manuscript is sectioned as follows: In Section 2, we have explained the concept of question difficulty and how it can be applied using a machine learning approach for readjusting the new difficulty levels of questions. Section 3 introduces the overall data pipeline process and the construction of the machine-learning model and describes the evaluation metrics we used for assessing the results of our experiment. Section 4 describes the results of feature selection based on statistical analysis. We have also described the results of the model performance based on several machine-learning models. In Section 5, the conclusions are presented.

## Related works

### Difficulty level of questions

Many previous studies have explored the concept of item difficulty levels. According to one study, item difficulty level is defined as the percentage of students who choose the correct answer out of all other students, as well as a scale that measures the degree of difficulty experienced by students when solving a set of problems. The level of question difficulty can be affected by the characteristics of the question or the type of learning or problem-solving model used [11, 12].

Other researchers have differentiated difficulty levels by examining socio-psychological perspectives. For example, Kim [7] has explained that question difficulty levels can be classified into two types, which can be differentiated based on the instructor’s expectations and the perspectives of learners regarding their feelings when answering the question. Specifically, they can be divided based on the instructors’ level of expectation; if most students can solve the problem within a given time, it would be easy, whereas if more time were taken, it would be hard. This possible difference in difficulty levels was explained through the authors’ experiment.

Similarly, Watering and Rijt [13] inferred that the standards for evaluating question difficulty levels differed between teachers and students. Their experimental results showed that most teachers overestimated the difficulty of most items by focusing only on a small fraction of items from the complete set of questions, excluding other factors involved, such as the current levels of the students’ knowledge and the skills they had acquired after taking the teacher’s course. Therefore, they diverged from the students’ point of view when they underestimated their own performance [13].

There is currently a trend toward using artificial intelligence to classify difficulty levels instead of the socio-psychological methods conventionally used. In other words, learning patterns can be analyzed by identifying learners’ behaviours through a quantitative analysis of questions. Zhao et al. [14] proposed and explained the use of a Markov-based model to categorize learning patterns into two types: volume-oriented learning (based on mass-oriented learning) and topic-oriented learning (based on topic-oriented learning). Mathematics can be viewed as a common language as it only requires basic numbers and operators to form equations, unlike linguistic subjects such as English, Chinese, and Spanish in which each language has different forms of dialects and grammars that must be followed accordingly. However, it is difficult to manually evaluate the mathematical ability of each learner according to their skill, knowledge, attitude, type of question, difficulty, and solution time. Therefore, to provide suitable mathematical questions for each learner, it is necessary to select a criterion for classifying such questions and design a new system that can automatically provide the next appropriate mathematical questions. Accordingly, in this study, correlation analysis was performed through a statistical approach to examine the factors that would play a significant role in classifying the difficulty level of questions.

### Supervised learning

The scope of machine learning can be broken down into three main sub-fields, namely supervised learning, unsupervised learning, and reinforcement learning, each of which plays an important role in the field; therefore, when learning the basics of artificial intelligence, each should be thoroughly explored in equal measure. For our analysis, we focused on using the supervised learning approach. In supervised learning, a trained model is used to learn multiple features that can be classified into multiple classes or labels from the observed data; then, a new label value is predicted based on the prediction value that most accurately matches the actual target variable.

In most recent research, machine learning approaches have been used to solve multi-classification problems; these approaches have shown excellent performance in various domains. In a study by Petersen and Ostendorf [15], the difficulty level on a reading proficiency test for elementary school students was evaluated through a machine-learning approach. After experimenting with and fitting the data using various language models (i.e., lexical-only, skip-gram, and non-syntactic approaches), each language model was compared with the baseline SVM model. The comparison of model performances revealed that the best performance was shown when all the language models, instead of just a single SVM model, were used [15]. Sangodiah, Ahmad, and Ahmad [16] also conducted a study on automatically classifying question difficulty levels by applying the text mining technique of term-frequency–inverse document frequency (tf–idf) [17] and the SVM model. Additionally, Alammary [18] established an effective evaluation system called LOsMonitor, which uses a combination of machine learning and text mining to classify the cognitive level of a learner’s evaluation questions and learning results. Although the supervised learning method has been used in many previous studies, there are few studies on the classification of mathematical questions according to their difficulty level. In this study, we intensively analyzed feature extraction and classified question difficulty levels through various machine learning techniques.

## Materials and methods

### Data pipeline process

An LMS is an online solution platform that manages learners and evaluates their skills through various online lecture courses. In our study, we analyzed the dataset provided by our collaborator ABLE Tech, a mathematical LMS-based solution platform targeting teenagers from elementary to high school.

**Fig 1:**
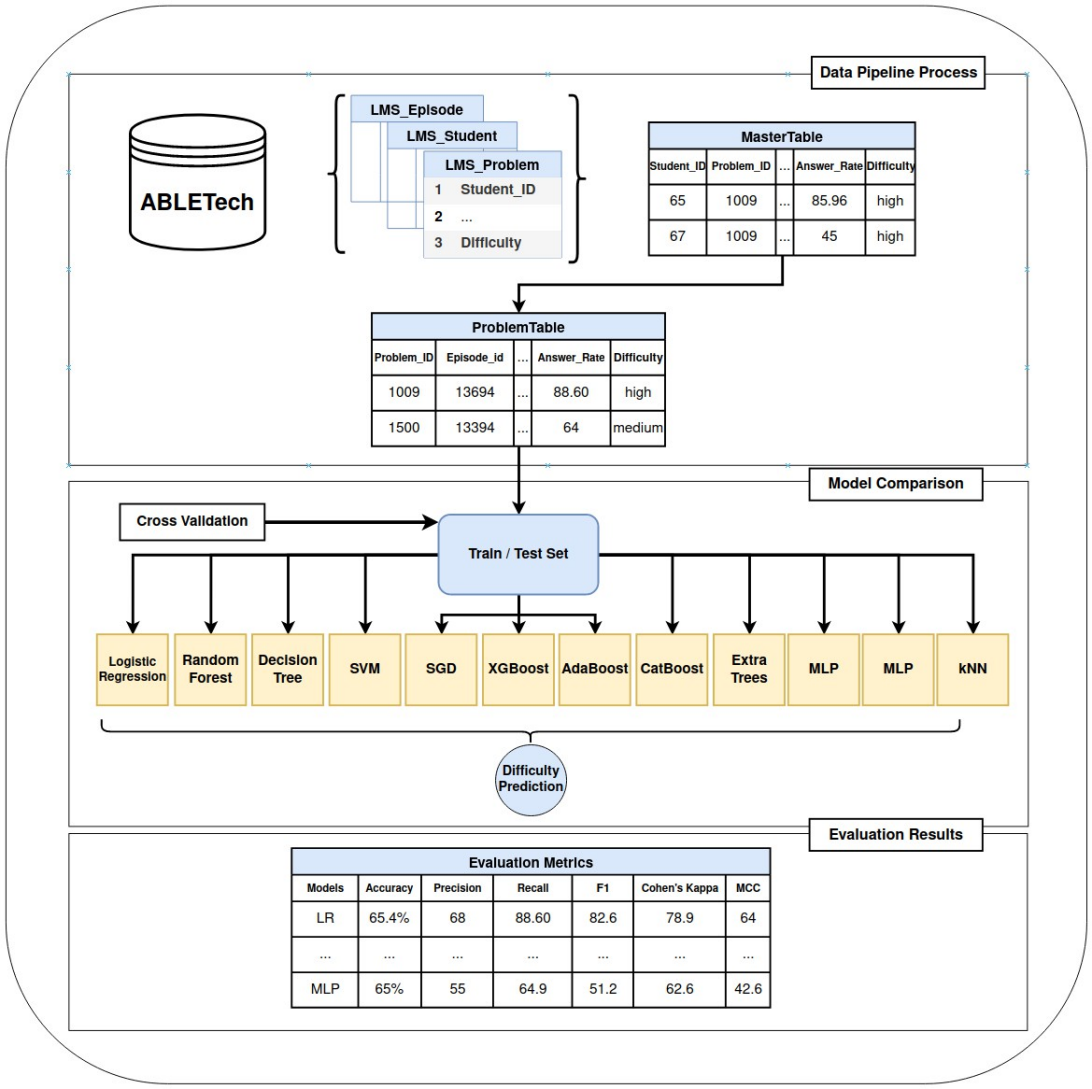
Overall workflow of math item classification system architecture. SVM: support vector machine, SGD: stochastic gradient descent, XGBoost: extreme gradient boosting, AUC-ROC: Area under the receiver operating characteristic curve, LMS: Learning management system.

The workflow system architecture used in this study, which consists of two parts, is shown in Fig 1. The first part shows the construction of the master table using a data pipeline; basic student information needs to be matched with information on the mathematical question for each student. A lot of processing time is required to build a master table with the various pieces of data provided by ABLE Tech.

When constructing the master table, certain students were excluded because information on them was unavailable. Initially, the provided data comprised 8.15 million rows; after preprocessing, only 3.12 million rows were used for the actual analysis. Additionally, it comprised information on students of various grades who responded to each mathematical question based on a unique ID value (i.e., problem ID). Since there were various grades of students for each question, the standard solution time could also be extracted as a new variable. In this study, the average and median solution times were compared and applied to a new variable as the median value, meaning that the question that was solved most frequently could be obtained using the problem ID. In the table wherein the questions have been sorted according to this frequency, the rate of correct answers that students solved for each problem ID could also be calculated as a new variable. The rate of correct answers refers to the percentage of students who correctly answered each question; each question had been divided into three components, namely chapter, episode (i.e., Episode ID), chapter (i.e., Chapter ID), and chapter type (i.e., Chapter type ID). The response options for the mathematical questions were largely divided into subjective and multiple-choice types. For the target variable, the difficulty of the existing labelled question was re-labelled to “high”, “medium”, or “low” as a multi-category for analysis, where “high” and “low” mean that the item is of high and relatively low difficulty, respectively. The seven final variables used in this study were the rate of correct answers, major chapter, intermediate chapter, small chapter, solution time, type of correct answer, and difficulty. The final data sample is shown in Table 1.

**Table 1:** Samples of final master table and main variables.

Problem ID	Answer rate	Episode ID	Chapter ID	Chapter type ID	Solution time	Answer type	Difficulty (Binary)	Difficulty (Multi)
65	81.69	50	59	252	37	0	low	low
66	59.21	50	59	252	77.5	0	high	high
67	84.06	50	59	252	44	0	low	low
73	42.31	53	65	271	18	1	high	high
149	45.57	50	59	252	42	0	low	medium

### Machine learning approach

In this study, we examined and analyzed the multi-class classification problem in relation to the level of difficulty in solving mathematical problems. Instead of using all the various classifier models, we focused on using optimal machine learning algorithms that had been employed in related research and were well-suited to our field of research. The following models were implemented and tested: logistic regression (LR), k-nearest neighbors algorithm (kNN), stochastic gradient descent (SGD), classification and regression tree (CART), support vector machine (SVM), multi-layer perceptron (MLP), random forest (RF), extra trees ensemble classifier (ET), adaboost (ADA), categorical boosting (CatBoost), gradient boosting machine (GBM), light gradient boosting machine (LightGBM), and extreme gradient boosting machine (XGB).

#### Logistic regression

Logistic regression (LR) is a statistical method that models the relationship between a dependent variable (binary outcome) and one or more independent variables (predictors). The dependent variable is represented as a binary variable (0 or 1) and is modelled using a logistic function. The logistic function transforms the linear combination of the independent variables into a probability value between 0 and 1. The following model can be represented mathematically as follows:

$$\begin{array}{c} p\left(y=1|x\right)=\frac{1}{1+e^{-z}} \end{array}$$ (1)

where *p*(*y* = 1|*x*) represents the probability of the binary outcome (y = 1) given the independent variables (x), and *z* = *b*_0_ + *b*_1_*x*_1_ + *b*_2_*x*_2_ + … + *b*_*n*_*x*_*n*_ represents the linear combination of the independent variables (x) and their coefficients (b), where *b*_0_ is the intercept and *b*_1_ to *b*_*n*_ are the coefficients of the independent variables *x*_1_ to *x*_*n*_. The function *e* is the base of the natural logarithm. The logistic function on the right-hand side of the equation (1/(1 + *e*^−*z*^)) maps the linear combination of the independent variables (*z*) to a probability value between 0 and 1, which represents the predicted probability of the binary outcome (*y* = 1) given the independent variables (*x*). The logistic function is also called the sigmoid function, and it has an S-shaped curve that is useful for modelling probabilities.

#### K-nearest neighbor

The k-Nearest Neighbor (kNN) algorithm is a simple and popular machine learning model used for classification and regression tasks. The algorithm works by finding the k closest data points in the training set to the input data point and predicting the output based on the majority vote of the k neighbors for classification, or the average value of the k neighbors for regression. The kNN model defines the distance between two data points as a metric function, such as the Euclidean distance or Manhattan distance. The prediction of the model can be written as:

$$\begin{array}{c} \hat{y}={\text{argmax}}_{y_{i}}\sum_{i=1}^{k}I\left(y_{i}=y\right) \end{array}$$ (2)

where $\hat{y}$ is the predicted output, *y*_*i*_ is the output of the *i*-th neighbor, and I is the indicator function that returns 1 if the condition inside the brackets is true, and 0 otherwise. The kNN algorithm has some drawbacks, such as the sensitivity to the choice of k and the computational complexity of finding the nearest neighbors in high-dimensional spaces. However, it remains a popular model for simple classification and regression tasks and can be useful as a baseline model for comparison with more complex models.

#### Stochastic gradient descent

Stochastic Gradient Descent (SGD) is an iterative optimization algorithm used to minimize the cost function in machine learning models. It works by updating the model parameters for each training example, unlike batch gradient descent which updates parameters based on the entire training dataset. SGD is more efficient and less computationally expensive than batch gradient descent and is widely used in deep learning models. The mathematical equation for updating the parameters in SGD can be represented as:

$$\begin{array}{c} \theta =\theta -\eta \nabla J(\theta ;x^{\left(i\right)},y^{\left(i\right)}) \end{array}$$ (3)

where *θ* are the model parameters, *η* is the learning rate, *J*(*θ*; *x*^(*i*)^, *y*^(*i*)^) is the cost function for a single training example (*x*^(*i*)^, *y*^(*i*)^), and ∇*J*(*θ*; *x*^(*i*)^, *y*^(*i*)^) is the gradient of the cost function with respect to the model parameters for a single training example. The update is performed for each training example in the dataset.

#### Decision tree

Classification and Regression Trees (CART) is a decision tree algorithm used for both regression and classification problems. It creates a binary tree structure where each internal node corresponds to a decision based on a feature and each leaf node corresponds to a prediction. The algorithm recursively partitions the data into smaller subsets based on the best split at each node. The splitting criterion used in CART is the reduction in variance or the Gini index for regression and classification problems, respectively. The optimal split is chosen based on the criterion that maximizes the reduction in variance or the Gini index. The algorithm stops when a stopping criterion is met, such as reaching a maximum tree depth or having too few samples at a node. Mathematically, the CART algorithm seeks to minimize the mean squared error (MSE) for regression problems:

$$\begin{array}{c} MSE=\frac{1}{N}\sum_{i=1}^{N}{\left(y_{i}-{\hat{y}}_{i}\right)}^{2} \end{array}$$ (4)

and the Gini index for classification problems:

$$\begin{array}{c} G=1-\sum_{i=1}^{K}p_{i}^{2} \end{array}$$ (5)

where *y*_*i*_ is the true target value for the *i*-th sample, ${\hat{y}}_{i}$ is the predicted value of the decision tree for that sample, K is the number of classes, and *p*_*i*_ is the proportion of samples that belong to the *i*-th class in the subset of data at that node.

#### Support vector machine

Support Vector Machines (SVM) is a machine learning algorithm used for classification and regression problems. The algorithm tries to find the best hyperplane that separates the data into different classes while maximizing the margin between the hyperplane and the nearest data points of each class. Mathematically, given a set of training data (*x*_*i*_, *y*_*i*_), where *x*_*i*_ is a feature vector and *y*_*i*_ is the corresponding target class label, it seeks to find the hyperplane *w*^*T*^ x + b = 0 that separates the data into two classes:

$$\begin{array}{c} f\left(x\right)=\text{sgn}\left(w^{T}x+b\right) \end{array}$$ (6)

where sgn is the sign function, and w and b are the model parameters that need to be learned from the data. The hyperplane is chosen to maximize the margin, which is the distance between the hyperplane and the nearest data points of each class. The optimization problem can be formulated as:

$$\begin{array}{c} \underset{w,b}{\text{min}}\frac{1}{2}{\left||w|\right|}^{2} \end{array}$$ (7)

subject to:

$$\begin{array}{c} y_{i}\left(w^{T}x_{i}+b\right)\geq 1,\forall i \end{array}$$ (8)

where ||*w*|| is the L2-norm of the weight vector, and the constraint ensures that the data is correctly classified and lies outside the margin. It is known for its ability to handle high-dimensional data and non-linear decision boundaries through the use of kernel functions. However, it can be sensitive to the choice of hyper-parameters and is not suitable for large datasets.

#### Multi-layer perceptron

A Multi-Layer Perceptron (MLP) is a feed-forward artificial neural network that consists of multiple layers of nodes, where each node in one layer is connected to every node in the subsequent layer. The nodes in the MLP are called neurons, and they apply a non-linear transformation to the input data. Mathematically, given a set of input data x, the output of the MLP is calculated as follows:

$$\begin{array}{c} f\left(x\right)=\sigma_{k}\left({\text{W}}_{k}\sigma_{k-1}\left({\text{W}}_{k-1}\cdots \sigma_{1}\left({\text{W}}_{1}x+{\text{b}}_{1}\right)\cdots +{\text{b}}_{k-1}\right)+{\text{b}}_{k}\right) \end{array}$$ (9)

where f(x) is the output of the MLP, *σ*_*i*_ is a non-linear activation function applied to the output of the *i*-th layer, *W*_*i*_ and *b*_*i*_ are the weight matrix and bias vector of the *i*-th layer, and k is the number of layers in the MLP. The MLP is trained using a supervised learning approach, where the weights and biases are adjusted to minimize the error between the predicted output and the actual output. This is typically done using a loss function such as mean squared error (MSE) or cross-entropy. MLPs are commonly used for classification and regression problems, and their performance can be improved by increasing the number of layers, adding regularization techniques, or using different activation functions. However, they can be sensitive to the choice of hyper-parameters and prone to over-fitting if the number of parameters is too large.

#### Random forest

Random forest (RF) is an ensemble learning method that combines multiple decision trees to make predictions. It is used for both regression and classification problems, and is known for its high accuracy and robustness to noise and over-fitting. The output of the algorithm is the average or majority vote of the individual trees. Mathematically, the output of a Random Forest model for a new sample x is:

$$\begin{array}{c} RF\left(x\right)=\{\begin{array}{cc} \frac{1}{T}\sum_{t=1}^{T}f_{t}\left(x\right) & \text{Regression}\\ \text{mode}(f_{1}\left(x\right),f_{2}\left(x\right),\ldots ,f_{T}\left(x\right)) & \text{Classification} \end{array} \end{array}$$ (10)

where T is the number of decision trees, and *f*_*t*_(*x*) is the prediction of the *t*-th decision tree for the input x. The randomness introduced in Random Forest helps to reduce over-fitting and improve the accuracy of the model.

#### Extra trees classifier

Extra Trees (ET) ensemble classifier is a machine learning algorithm that combines multiple decision trees to create a predictive model. ET builds on the Random Forest algorithm, but with some key differences. Like Random Forest, ET creates multiple decision trees using bootstrap samples of the training data and random subsets of the features. However, it also selects the splitting point at each node of the tree using a random threshold value, rather than finding the best threshold. This randomization can result in less bias and variance in the model, and can improve the accuracy. The mathematical equation for ET is similar to that of a decision tree ensemble:

$$\begin{array}{c} F\left(x\right)=\sum_{i=1}^{n}f_{i}\left(x\right) \end{array}$$ (11)

where *F*(*x*) is the model’s prediction for a new input, *n* is the number of trees in the ensemble, and *f*_*i*_(*x*) is the prediction of the *i*th decision tree in the ensemble. Each decision tree is built using a random subset of the features and a random threshold value for each split. The final prediction is determined by averaging the predictions of all the trees in the ensemble.

#### Adaptive boosting

AdaBoost (Adaptive Boosting) is a popular boosting algorithm that combines multiple weak classifiers to form a strong classifier. The basic idea behind AdaBoost is to iteratively train weak classifiers on the training data, and assign higher weights to the misclassified samples at each iteration. This allows the subsequent weak classifiers to focus more on the previously misclassified samples. Mathematically, the AdaBoost algorithm can be expressed as follows:

$$\begin{array}{c} F\left(x\right)=\sum_{t=1}^{T}\alpha_{t}f_{t}\left(x\right) \end{array}$$ (12)

where *F*(*x*) is the final strong classifier, *f*_*t*_(*x*) is the weak classifier at iteration *t*, and *α*_*t*_ is the weight assigned to the weak classifier. The weight *α*_*t*_ is calculated based on the performance of the weak classifier on the training data, and is higher for more accurate classifiers. During training, AdaBoost assigns initial weights to each sample in the training set. These weights are then updated at each iteration based on the accuracy of the weak classifier on the training data. The final output of the strong classifier is determined by combining the weighted outputs of the weak classifiers. AdaBoost is a powerful algorithm that can achieve high accuracy with relatively few weak classifiers. However, it can be sensitive to outliers and noise in the data.

#### Categorical boosting

Categorical boosting (CatBoost) is a gradient boosting algorithm that is designed to handle categorical features in machine learning problems. It uses a combination of ordered boosting, categorical features processing, and gradient-based decision tree algorithms to achieve high accuracy with categorical data. CatBoost also includes a set of techniques to prevent over-fitting and handle missing data. The mathematical equation for CatBoost is similar to other gradient boosting algorithms:

$$\begin{array}{c} F_{m}\left(x\right)=F_{m-1}\left(x\right)+\gamma_{m}h_{m}\left(x\right) \end{array}$$ (13)

where *F*_*m*_(*x*) is the model’s prediction at iteration *m*, *F*_*m*−1_(*x*) is the prediction at iteration *m* − 1, *γ*_*m*_ is the learning rate for iteration *m*, and *h*_*m*_(*x*) is the weak model at iteration *m*. However, CatBoost uses a unique algorithm for handling categorical data, which involves splitting the categorical features into ordered groups and encoding them as numerical values. This approach can result in more accurate and efficient models for datasets with categorical features.

#### Gradient boosting variants

Gradient Boosting Machine (GBM) is a machine learning algorithm that creates a predictive model by combining multiple weak models. GBM works by iteratively fitting new models to the residuals of the previous models, and combining their predictions to improve the overall accuracy. GBM is a powerful algorithm that can handle a variety of data types and is commonly used for regression and classification problems. The mathematical equation for GBM can be represented as:

$$\begin{array}{c} F_{m}\left(x\right)=F_{m-1}\left(x\right)+\gamma_{m}h_{m}\left(x\right) \end{array}$$ (14)

where *F*_*m*_(*x*) is the model’s prediction at iteration *m*, *F*_*m*−1_(*x*) is the prediction at iteration *m* − 1, *γ*_*m*_ is the learning rate for iteration *m*, and *h*_*m*_(*x*) is the weak model at iteration *m*. The weak model is usually a decision tree that predicts the residuals of the previous model. The GBM algorithm iteratively adds new weak models to the ensemble until the desired level of accuracy is achieved.

Similar to the gradient boosting machine (GBM) framework, LightGBM is the lightweight version of the gradient boosting mechanism and it is designed to be highly efficient and scalable, making it well-suited for large-scale machine learning applications. LightGBM uses histogram-based algorithms for computing gradients, which reduces memory usage and computation time. It also uses a leaf-wise tree growth strategy, which can lead to better accuracy with fewer trees. The mathematical equation for LightGBM is similar to the standard gradient boosting algorithm. However, LightGBM also introduces a new feature called “leaf-wise growth,” which means that the tree is grown by splitting the leaf that results in the largest reduction in the loss function, rather than the traditional level-wise approach. This results in a more accurate and efficient model.

#### Extreme gradient boosting

Extreme Gradient Boosting (XGBoost) is a machine learning model that uses a gradient boosting framework to improve the performance of decision trees. The xgboost model is designed to handle large-scale datasets and is known for its speed, accuracy, and robustness. The xgboost model combines the outputs of multiple decision trees to make more accurate predictions. Each decision tree is trained on a subset of the data and the residuals of the previous trees, and the final prediction is made by aggregating the predictions of all the trees. The xgboost model uses a loss function to measure the difference between the predicted and actual values, and the gradient of the loss function is used to update the parameters of the model. The objective function of xgboost is defined as:

$$\begin{array}{c} L\left(\theta \right)=\sum_{i=1}^{n}l\left(y_{i},\hat{y_{i}}\right)+\sum_{k=1}^{K}\Omega \left(f_{k}\right) \end{array}$$ (15)

where *θ* represents the model parameters, l is the loss function, *y*_*i*_ is the actual value, $\hat{y_{i}}$ is the predicted value, K is the number of trees, and Ω(*f*_*k*_) is a regularization term that penalizes complex models. The main goal is to accurately predict the target variable based on a large number of features. Given the brief summary of each machine learning-based models, hyper-parameter tuning is important for achieving optimal performance under each machine learning method. Accordingly, we used GridSearchCV to automatically find the optimal parameter values using the Python-based scikit-learn package. GridSearchCV is a lattice search technique that solves the complexity of machine learning models and reduces the repetitive computation time required to find optimal parameter values [19–21]. Table 2 summarizes the optimal parameter values for each model used in this study.

**Table 2:** Optimal parameter value results obtained through grid-search cross-validation.

Models	Optimized parameters
LR	C : 64 solver : sag multi_class : multinomial
kNN	n_neighbors : 64
SGD	C : 1500 kernel : rbf gamma : 0.01 probability : True
CART	max_depth : 8
SVM	alpha : 0.005 learning_rate : optimal loss : modified_huber
MLP	alpha : 0.0005 solver : lbfgs learning_rate_init : 0.0001
RF	n_estimators : 1024 criterion : gini max_depth : 16
ET	criterion : gini
ADA	learning_rate : 1 n_estimators : 1024
CatBoost	default
GBM	loss : log_loss n learning_rate : 0.1
LightGBM	boosting_type : gbdt learning_rate : 0.1
XGB	learning_rate : 0.001 n_estimators : 512 max_depth : 100 subsample : 0.7 colsample_bytree : 0.7 objective : multi : softmax

LR: logistic regression, kNN: k-nearest neighbors algorithm, SGD: stochastic gradient descent, CART: classification and regression tree, SVM: support vector machine, MLP: multilayer perceptron, RF: random forest, ADA: Adaptive boosting, ET: extra trees ensemble classifier, CatBoost: categorical boosting classifier, GBM: gradient boosting machine, LightGBM: light gradient boosting machine, XGB: extreme gradient boosting machine.

### Evaluation metrics

To compare the model’s performances, different evaluation criteria were used. Accuracy was used as an index to evaluate the similarity of the predicted data with the actual data and thereby determine the accuracy of the model. The confusion matrix, which is used as a performance indicator in binary classification, is an indicator that shows the level of confusion in a trained classification model when calculating the predicted values. The one-vs-rest (OVR) method was used to calculate the confusion matrix in this study, and the results were used for multiple classification. In other words, after being divided into “high”, “medium”, or “low” based on the difficulty levels, the questions could be classified into two groups, such as “high” and “middle/low,” “middle” and “high/low,” and “low” and “high/middle,” and the final value would be calculated as the average value for each category. In the case of an unbalanced dataset, the reliability could be further improved by measuring the values of precision and recall, which have been recommended more than the accuracy score has in existing research. Precision and recall can be evaluated intensively for the predictive performance of positive datasets by using the true-positive (TP), false-positive (FP), and false-negative (FN) values calculated from the confusion matrix. The precision and recall values were calculated using the following equations:

$$\begin{array}{c} \text{Precision}=TP/(FP+TP), \end{array}$$ (16)

$$\begin{array}{c} \text{Recall}=TP/(FN+TP), \end{array}$$ (17)

The F1 score is an index combining both precision and recall. This index is used to evaluate the balance between precision and recall; the closer it is to 1, the better the performance. Finally, the AUC-ROC curve was used to evaluate the model’s performance. Using this curve, the change in TP ratio (value predicted as true among true) and the change in FP ratio (value predicted as false among false) can be visually evaluated. In addition, due to the imbalanced dataset in terms of difficulty levels, comparing our models with accuracy and F1 scores may not be sufficient enough to justify which model is the best model. In this case, we additionally used two statistical methods of Cohen’s Kappa and Matthew’s Correlation Coefficient (MCC) metrics to further investigate and analyze our model comparisons. Cohen’s Kappa measures the agreement between two raters (i.e., the model and the ground truth labels) by accounting for the agreement that could occur by chance. The formula for Cohen’s Kappa is as follows:

$$\begin{array}{c} K=\frac{p_{o}-p_{e}}{1-p_{e}} \end{array}$$ (18)

where *p*_*o*_ is the observed proportion of agreements between the raters and *p*_*e*_ is the proportion of agreements expected by chance. *p*_*o*_ is calculated as the number of observed agreements between the raters divided by the total number of ratings, while *p*_*e*_ is calculated as the product of the marginal proportions of each rater’s categories. It ranges from -1 to 1, where values closer to 1 indicate higher agreement between the model and the ground truth labels. Also, MCC is another metric that takes into account the true positives, true negatives, false positives, and false negatives. MCC can be calculated by using the concepts of confusion matrix metrics. The following equation computes the MCC score:

$$\begin{array}{c} MCC=\frac{TP\times TN-FP\times FN}{\sqrt{\left(TP+FP)(TP+FN)(TN+FP)(TN+FN\right)}} \end{array}$$ (19)

where TP is the number of true positives, TN is the number of true negatives, FP is the number of false positives, and FN is the number of false negatives. MCC ranges from -1 to 1, where values closer to 1 indicate better performance. Both Cohen’s Kappa and MCC can be useful in model comparisons because they take into account both the accuracy and the balance between the different classes.

## Results

The difficulty levels of mathematical questions can be evaluated on a numerical or character basis. In this study, the difficulties of the questions were classified based on the characteristics of the questions. Given the provided data, the values in the “multi-difficulty” variable for each question ranged from 0 to 10; these were converted and labelled as “high,” “medium,” or “low” based on the given range of scores.

### Exploratory data analysis

The data on the mathematical questions used for the multi-class classification problem were obtained from 2015 to 2022, and they comprised approximately 320,000 rows. In this study, there were about 17,050 questions grouped by question IDs (i.e., problem ID), and the grade range of students was from the 3rd grade of elementary school to the 2nd grade of high school; elementary, middle, and high school students accounted for 51.4, 47.8, and 0.83% of the total number of students, respectively, with there being more elementary and middle school students than high school students. Accordingly, there were more questions corresponding to elementary and middle school students; brief information on the variables used in the experiment is shown in Table 3.

**Table 3:** Data on students.

Grades	Number of students	Analyzed datasets
Elementary	184	17,050
Middle school	171	
High school	3	

### Feature selection

A statistical t-test analysis was performed to verify the results of feature selection between the candidate variables. Variables other than answer type influenced question difficulty among the independent variables. Variables other than answer type were significant at a significance level of ±5%, and all the p-values were smaller than 0.05. The results of the significance tests are presented in Table 4.

**Table 4:** Indices for statistical t-test analysis results.

Features	t-score	P > |*t*|	Support
Answer rate	14.310	>0.000	Yes
Episode	38.584	>0.000	Yes
Chapter	27.813	>0.000	Yes
Chapter type	12.049	>0.000	Yes
Solution time	19.988	>0.000	Yes
Answer type	1.094	0.274	No

### Discussion

**Fig 2:**
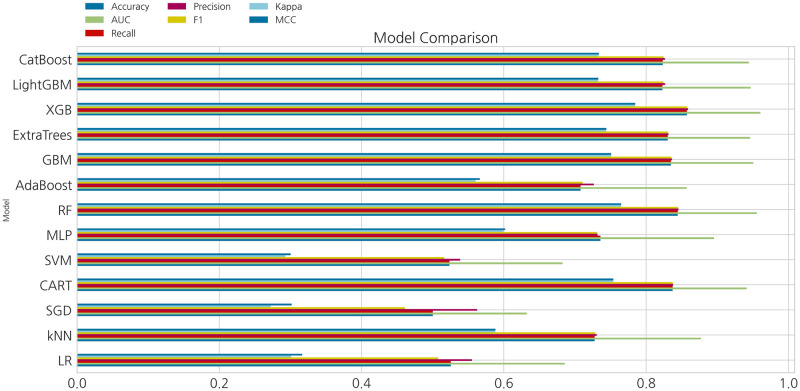
Evaluation metrics of model performances.

**Fig 3:**
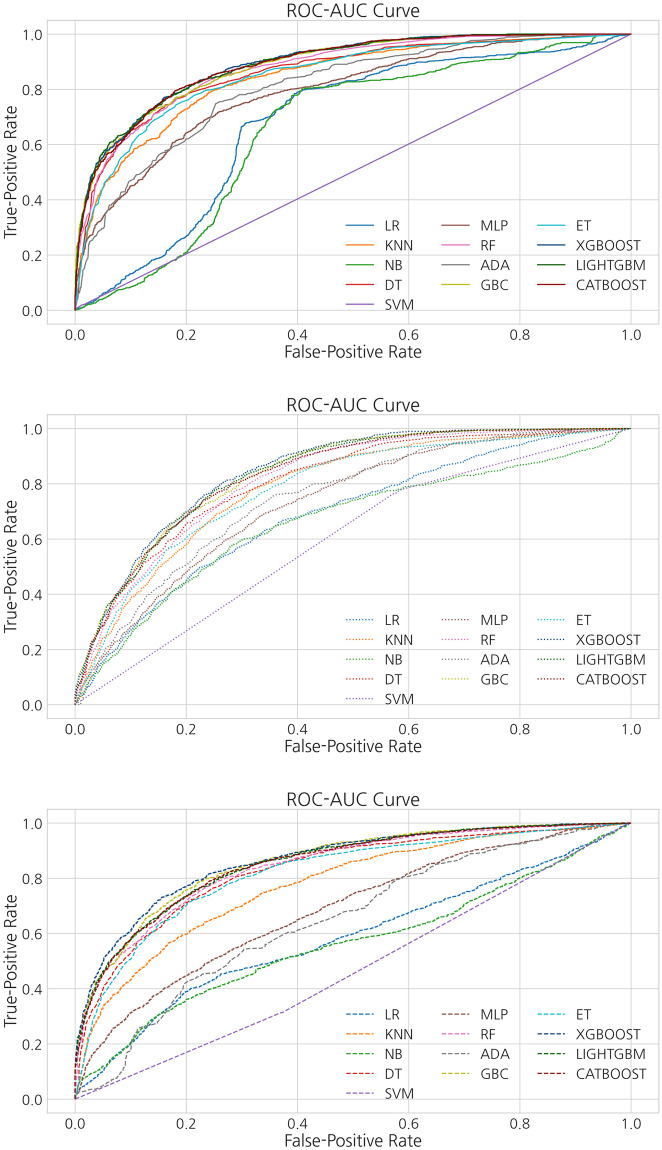
AUC-ROC curve for each difficulty level, namely “high”, “medium”, and “low”.

Fig 2 shows the results of comparing the performance of various machine learning models based on the criteria for classifying the difficulty of each mathematical question. In this study, seven evaluation indicators were used to increase the accuracy and reliability of the results. In terms of the conventional statistical methods of logistic regression and SVM, it has shown weak performances in overall metrics where logistic regression and SVM performed 52.5% and 52.3% in accuracy, 0.507 and 0.516 in F1 score, and 0.3 and 0.293 in Kappa metrics respectively. These statistical methods showed weak performances as they assume linear relationships between the features and the target variable, which limits their ability to model complex non-linear relationships. In this sense, the more features we consider, the more complex it becomes to classify our target variable. In addition, conventional statistical methods are sensitive to outliers which can drastically affect the performance to drop dramatically. In terms of the clustering method of kNN, it performed strong in accuracy and F1 scores of 72.7% and 0.728, but weak in Kappa and MCC metrics with 0.588. kNN model can perform well in terms of accuracy and F1 score but be weak in Kappa and MCC metrics if the classes are imbalanced or if the model tends to make errors in specific types of instances, leading to a lower agreement between the predicted and true labels. Other models that are based on decision tree algorithms, such as CART, random forest, and extra trees classifier have outperformed the conventional statistical methods in all metrics. Also, gradient boosting variants, such as catboost, GBM, and lightGBM, performed 82.3%, 83.5%, and 82.3% in accuracy, 0.825, 0.836, and 0.824 in F1 score, and 0.945, 0.951, and 0.947 in AUC-ROC curve metrics respectively. During our experiment, the xgboost model showed the best performance in terms of accuracy, with an accuracy of approximately 85.7%. Additionally, when comparing the precision and recall values among the models, this model showed precision and recall values of 85.9 and 85.7%, respectively. The F1 score, which evaluates overall precision and recall, was also evaluated. Based on these results, it was inferred that the xgboost model outperformed the other models. Table 5 presents a comparison of the values of the evaluation indicators for each model. Additionally, in this experiment, the evaluation of the AUC-ROC curve score was used in determining the classification performance for questions of each level of difficulty. The TP ratio was evaluated using the AUC-ROC curve. A visual comparison of the models through the AUC-ROC curves is shown in Fig 3. Specifically, each figure shows the “high”, “medium”, and “low” of the TP rates for each difficulty level of questions. Comparing the scores of the AUC-ROC curve, the xgboost model outperformed the other machine learning models with a performance of 96.1%. Compared to the conventional statistical and artificial neural network based models, the advantages of xgboost is dependent on the mechanism that it works by iteratively adding new decision trees that correct the errors made by the previous trees, resulting in a highly accurate model. Also, gradient boosting can handle non-linear relationships which can capture complex interactions and non-linearities, and provides a measure of feature importance, which helps in identifying the most relevant features for the classification task. This mechanism allows the model to simplify, reduce over-fitting problem, and improve interpretability.

**Table 5:** Comparison of evaluation indicators of different machine-learning models.

Models	Accuracy	Precision	Recall	F1	AUC-ROC	Kappa	MCC
LR	0.525	0.555	0.525	0.507	0.686	0.301	0.316
kNN	0.728	0.731	0.728	0.729	0.877	0.588	0.588
SGD	0.501	0.562	0.501	0.461	0.632	0.272	0.302
CART	0.837	0.838	0.837	0.838	0.941	0.754	0.754
SVM	0.523	0.539	0.523	0.516	0.683	0.293	0.300
MLP	0.736	0.732	0.736	0.731	0.895	0.599	0.602
RF	0.844	0.846	0.844	0.845	0.956	0.765	0.765
ET	0.830	0.831	0.830	0.831	0.946	0.744	0.744
ADA	0.708	0.727	0.708	0.711	0.857	0.560	0.566
CatBoost	0.823	0.826	0.823	0.825	0.945	0.733	0.734
GBM	0.835	0.837	0.835	0.836	0.951	0.751	0.751
LightGBM	0.823	0.827	0.823	0.824	0.947	0.733	0.733
**XGB**	**0.857**	**0.859**	**0.857**	**0.858**	**0.961**	**0.784**	**0.785**

LR: logistic regression, kNN: k-nearest neighbors algorithm, SGD: stochastic gradient descent, CART: classification and regression tree, SVM: support vector machine, MLP: multilayer perceptron, RF: random forest, ET: extra trees classifier, ADA: adaptive boosting, CatBoost: categorical boosting, GBM: gradient boosting machine, LightGBM: light gradient boosting machine, XGB: extreme gradient boosting

## Conclusion

In this study, considering the characteristics of various items, we attempted to classify item difficulties. A statistical t-test analysis was conducted to verify whether the characteristics of each item had a significant effect on its difficulty. The analysis revealed a positive correlation between the rate of correct answers for the items, each section of the item (i.e., episode, chapter, and chapter type units), and the solution time. However, there was no correlation with the answer type of the item; therefore, this characteristic was excluded from our analysis. The rate of correct answers for each item, each section of the item, and the solution time had p-values less than 0.05, supporting the assumptions in our experiment. Additionally, the difficulty of the mathematical question items was classified using various machine learning techniques. The xgboost model showed superior performance to those of statistical machine learning techniques, such as logistic regression, support vector machine, random forest, decision tree-based models, and k-nearest neighbor models. The xgboost model also had the best accuracy, F1, AUC-ROC, Kappa and MCC of 85.7%, 0.858, 0.961, 0.784 and 0.785 respectively.
